# Lenalidomide normalizes tumor vessels in colorectal cancer improving chemotherapy activity

**DOI:** 10.1186/s12967-016-0872-2

**Published:** 2016-05-05

**Authors:** V. Leuci, F. Maione, R. Rotolo, E. Giraudo, F. Sassi, G. Migliardi, M. Todorovic, L. Gammaitoni, G. Mesiano, L. Giraudo, P. Luraghi, F. Leone, F. Bussolino, G. Grignani, M. Aglietta, L. Trusolino, A. Bertotti, D. Sangiolo

**Affiliations:** Department of Oncology, University of Torino, Turin, Italy; Laboratory of Medical Oncology-Experimental Cell Therapy, Candiolo Cancer Institute-FPO- IRCCS, Candiolo Turin, Italy; Division and Laboratory of Medical Oncology, Candiolo Cancer Institute-FPO- IRCCS, Candiolo Turin, Italy; Laboratory of Transgenic Mouse Models, Candiolo Cancer Institute-FPO- IRCCS, Candiolo Turin, Italy; Laboratory of Translational Cancer Medicine, Candiolo Cancer Institute-FPO- IRCCS, Candiolo Turin, Italy; Laboratory of Vascular Oncology, Candiolo Cancer Institute, Candiolo Turin, Italy; Laboratory of Cancer Stem Cell Research, Candiolo Cancer Institute-FPO- IRCCS, Candiolo Turin, Italy; Department of Science and Drug Technology, University of Torino, Turin, Italy

**Keywords:** Lenalidomide, Colorectal cancer, Tumor-vessel normalization

## Abstract

**Background:**

Angiogenesis inhibition is a promising approach for treating metastatic colorectal cancer (mCRC). Recent evidences support the seemingly counterintuitive ability of certain antiangiogenic drugs to promote normalization of residual tumor vessels with important clinical implications. Lenalidomide is an oral drug with immune-modulatory and anti-angiogenic activity against selected hematologic malignancies but as yet little is known regarding its effectiveness for solid tumors. The aim of this study was to determine whether lenalidomide can normalize colorectal cancer neo-vessels in vivo, thus reducing tumor hypoxia and improving the benefit of chemotherapy.

**Methods:**

We set up a tumorgraft model with NOD/SCID mice implanted with a patient-derived colorectal cancer liver metastasis. The mice were treated with oral lenalidomide (50 mg/Kg/day for 28 days), intraperitoneal 5-fluorouracil (5FU) (20 mg/Kg twice weekly for 3 weeks), combination (combo) of lenalidomide and 5FU or irrelevant vehicle. We assessed tumor vessel density (CD146), pericyte coverage (NG2; alphaSMA), in vivo perfusion capability of residual vessels (lectin distribution essay), hypoxic areas (HP2-100 Hypoxyprobe) and antitumor activity in vivo and in vitro.

**Results:**

Treatment with lenalidomide reduced tumor vessel density (p = 0.0001) and enhanced mature pericyte coverage of residual vessels (p = 0.002). Perfusion capability of tumor vessels was enhanced in mice treated with lenalidomide compared to controls (p = 0.004). Accordingly, lenalidomide reduced hypoxic tumor areas (p = 0.002) and enhanced the antitumor activity of 5FU in vivo. The combo treatment delayed tumor growth (p = 0.01) and significantly reduced the Ki67 index (p = 0.0002). Lenalidomide alone did not demonstrate antitumor activity compared to untreated controls in vivo or against 4 different mCRC cell lines in vitro.

**Conclusions:**

We provide the first evidence of tumor vessel normalization and hypoxia reduction induced by lenalidomide in mCRC in vivo. This effect, seemingly counterintuitive for an antiangiogenic compound, translates into indirect antitumor activity thus enhancing the therapeutic index of chemotherapy. Our findings suggest that further research should be carried out on synergism between lenalidomide and conventional therapies for treating solid tumors that might benefit from tumor vasculature normalization.

## Background

Angiogenesis inhibition has shown promising therapeutic activity against metastatic colorectal cancer (mCRC). VEGF blockade by the monoclonal antibody bevacizumab is currently incorporated in clinical practice and other compounds with similar activity are being explored in clinical trials [1–8]. These approaches are based on the idea of starving tumors thus preventing or reducing neovasculature formation [9–11]. Recent evidences have demonstrated that another promising, and seemingly counterintuitive, aspect of some antiangiogenic compounds is the potential to promote the normalization of tumor vessels [12–15], which are typically immature and disorganized in tumors [10, 16]. This effect may have therapeutic benefit by enhancing tumor oxygenation, delivery of therapeutic compounds or even immune effector cells [17–19]. Abnormal tumor vasculature reduces blood flow into tumor sites, potentially hindering the delivery of chemotherapy drugs and thus promoting an aggressive hypoxic microenvironment that fuels a vicious circle which results in the upregulation of pro-angiogenic factors [20–23]. Improved chemotherapy outcome was associated with vessel normalization in preclinical tumor models [24–27]. Assessment of vessel normalization is often based on morphologic vessel remodeling with functional evidences of mature pericyte-coverage and restored perfusion ability with consequent variation in tissue oxygenation/hypoxia status [28–30]. Awareness of the importance of tumor-vessel normalization could modify and enrich the old idea of anti-angiogenic therapy. Further research is required to explore the effect of antiangiogenic compounds on vessel-normalization in settings such as mCRC, where antiangiogenesis is considered to be a significant clinical approach. Lenalidomide is an oral derivative of thalidomide, currently approved for treating selected hematologic malignancies such as multiple myeloma and 5q- myelodysplastic syndrome [31–35]. Although the antitumor mechanism of lenalidomide is not yet completely clear, it seems to induce angiogenesis inhibition and immunomodulation [36, 37]. Initial experimental trials are exploring the activity of lenalidomide in mCRC. These studies are aimed at exploiting the immunomodulation property of lenalidomide in order to increase the activity of anti-EGFR mAb cetuximab [38, 39].

We hypothesized that lenalidomide might exert an additional beneficial effect on antiangiogenic and tumor-vessel normalization activity.

Indeed although it is assumed, tumor-vessel normalization has not yet been effectively explored. We set a preclinical mCRC model based on a single patient-derived tumorgraft model generated from a sample of liver CRC metastasis [40, 41]. This model was shown to be representative of the original patient’s tumor. It helps to retain the actual tumor tissue architecture, thus providing a platform for studying issues such as tumor neo-vessels and their modulation in vivo. This model was used to explore the antiangiogenic and normalization effects of oral lenalidomide on CRC neo-vessels, including its potential to improve tumor perfusion, oxygenation and the therapeutic index of conventional chemotherapy.

## Methods

### Tumor cell lines

All colorectal cancer cell lines were obtained by means of American type culture collection (ATCC). Appropriate culture medium [HCT116 and LS513 grown in RPMI-1640 medium, SKCO-1 grown in DMEM medium, and LoVo grown in F-12 K medium; American type culture collection (ATCC)] was supplemented with 10 % FBS (Sigma), 100 U/mL penicillin and 100 U/mL streptomycin (Sigma) in a humidified 5 % CO2 incubator at 37 °C.

### Murine CRC patient-derived tumorgraft

All animal procedures were approved by the internal animal research ethical committee and the Italian Ministry of Health. The metastatic CRC sample was obtained by carrying out a liver metastasectomy on a patient treated at our center. Before the surgical intervention the patient was treated with four courses of neo-adjuvant chemotherapy with capecitabine and oxaliplatin. The tumor presented the following genetic mutations: KRAS (p.G12C), APC (p.K1370*) and TP53 (p.V173E). The patient provided informed consent and the study was conducted according to a protocol approved by the institutional review board. Tumor material not required for histopathologic analysis was collected and placed in medium 199 supplemented with 200 U/mL penicillin, 200 μg/mL streptomycin, and 100 μg/mL levofloxacin. Each sample was cut into 25- to 30 mm^3^ pieces in antibiotic-containing medium; some of the pieces were incubated overnight in RNA later and then frozen at −80 °C for molecular analyses; 2 other pieces were coated in Matrigel (BD Biosciences) and implanted in 2 different 4- to 6-week-old female NOD (nonobese diabetic)/SCID (severe combined immunodeficient) mice (Charles River). After mass formation, the tumors were passaged and expanded for 2 generations until production of a cohort of 32 mice [41, 42]. Four-week-old NOD/SCID mice with established tumors (average volume 400 mm3) were divided into four groups (8 mice per group) and treated with oral lenalidomide (kindly provided by Celgene) 50 mg/Kg/day for 28 days, intraperitoneal 5-Fluorouracile (5FU) (20 mg/Kg twice weekly for 3 weeks) from pharmacy leftovers, combination of lenalidomide and 5FU (doses as previously indicated) or irrelevant vehicle (0.5 % carboxymethylcellulose and 0.25 % polyoxyethylene sorbitan monooleate (Tween^®^ 80) in sterile water) respectively. Lenalidomide was administered to the mice via oral gavage at the above indicated dose in an aqueous suspension containing 0.5 % carboxymethylcellulose and 0.25 % Tween^®^ 80 at a dose volume of 5 mL/kg.

Tumor size was evaluated weekly by taking caliper measurements and the approximate volume of the mass was calculated with the formula 4/3 × π(d/2)2 × D/2, where d is the minor tumor axis and D is the major tumor axis. The tumor was fixed overnight in 4 % paraformaldehyde, dehydrated, paraffin-embedded, sectioned (5 μm) and stained with hematoxylin and eosin (H&E.; Bio Optica).

The percentage of tumor growth inhibition for each arm of treatment was calculated with the Eq. 100 − (T/C × 100), where T is the mean relative tumor volume (RTV) of the treated tumor and C is the mean RTV in the control group at the time of sacrifice.

The individual RTV was defined as Vx/V1, where Vx is the volume in mm3 at the end of the experiment and V1 at the start of treatment.

### Immunohistochemistry

Paraffin-embedded or OCT-embedded frozen samples were cut into 5-μm thick sections. The tissue slides were treated according to standard immunofluorescence or immunohistochemistry procedures. In short, the slides were permeabilized in 0.1 % Triton X-100 and 0.3 % Tween 20 (Sigma-Aldrich) in TBS, treated for 30 min with 1 % hydrogen peroxide to quench endogenous peroxidases in immunohistochemistry experiments, and saturated with 5 % goat serum (Sigma-Aldrich) in PBS. The slides were incubated with individual primary antibodies overnight at 4 °C inside a moist chamber. After rinsing in PBS, a secondary antibody was added. Secondary HRP-conjugate antibodies (EnVision; DakoCytomation) were used for immunohistochemistry and the reaction was visualized with DAB chromogen (DakoCytomation Liquid DAB Substrate Chromogen System, Dako). The tissues were counterstained with Mayer hematoxylin (Bio-Optica), mounted on glass slides and visualized with a BX-60 microscope (Olympus) equipped with a color Qicam Fast 1394-digital CCD camera (12 bit; QImaging). For carrying out the immunofluorescence experiments, Alexa Fluor secondary antibodies were used conjugated with Alexa Fluor 488 or Alexa Fluor 555 fluorochromes (Invitrogen). Molecular probes were used for counterstaining the nuclei with DAPI (Invitrogen). The slides were analyzed under a Leica DM IRBM microscope. The tissues were stained with the following primary antibodies: anti–Ki-67 (DAKO); Anti-MCAM [CD146] (clone EPR3208, Millipore); purified rat monoclonal anti-panendothelial cell ANTIGEN (Meca32) (550563, clone Meca32, 1:100, BD Pharmingen); rabbit polyclonal anti-α-SMA (AB5694, 1:100, Abcam).

### Tumor vasculature quantification

Tumor vasculature was evaluated by immunohistochemistry. For each animal, the total vessel area in each tumor section was quantified as CD146 positive structures by computer assisted analysis employing Image-ProPlus 6.2 software (Media Cybernetics). The CD146 positive surface area occupied by vessels was compared with total tissue area. In order to better account for the elevated intratumoral heterogeneity, at least 25 microscopic observational fields (MOF) (40× magnification) were recorded and analyzed from sections of 5 tumors for each experimental condition (40× magnification).

### Immunofluorescence analysis for pericyte coverage and tumor vessel perfusion

All immunofluorescence images were captured and analyzed with a Leica SPIIE confocal laser-scanning microscope (Leica Microsystems). Image acquisition was performed maintaining the same laser power, gain, and offset settings in order to quantify pericyte coverage (α-SMA and NG2 markers shown by red and green channels, respectively) and at least 16 multiple observational fields (40× magnification) from sections of 3 tumors for each experimental condition were recorded and analyzed. We drew a region of interest (ROI) close to each blood vessel (Meca32, shown by violet channels) and then quantified the MFI of red/green and violet channels using the LAS AF Quantification Tool. We then calculated the ratio between red/green and violet channels for each ROI. In this way the pericyte values were normalized on the vessel area. A similar procedure was used for quantifying vascular perfusion by FITC-labeled lectin (Vector laboratories, Inc.) shown by the green channel. The mice were injected i.v. with 0.05 mg FITC-labeled tomato lectin (Lycopersicon esculentum; Vector Laboratories) and were euthanized after 2 min. The lectin distribution was visualized by means of fluorescent confocal microscopy. The experiments included at least 16 MOF (40× magnification) from sections of 3 tumors for each experimental condition.

### Tumor hypoxia analysis

The amount of tumor hypoxia was determined 15 min after injecting 60 mg/kg pimonidazole hydrochloride (HP2-100 Hypoxyprobe Kit-Plus; Natural Pharmacia International Inc.) into NOD/SCID mice [43]. The formation of pimonidazole adducts was detected by immunostaining with Hypoxyprobe-1-Mab1 FITC Ab according to the manufacturer’s instructions. Immunofluorescence images were captured and analyzed with a Leica SPIIE confocal laser-scanning microscope (Leica Microsystems).

Immunostaining with Hypoxyprobe-1-Mab1 FITC Ab was quantified in at least 10 multiple observational fields (40× magnification) from sections of three tumors for each experimental condition. In order to quantify hypoxic areas in each image, we drew a Region of Interest (ROI) close to each green area (pimonidazole adducts, shown by green channel) and a ROI close to each viable tissue area (DAPI, shown by blue channels) and we then quantified the area with Image-ProPlus 6.2 software (Media Cybernetics). The ratio between the green and blue areas was calculated and the values were expressed as percentages.

### Cell viability assays

Four CRC cell lines (HCT116, LS513, SKCO-1 and LoVo cells) were plated at a density of 10 cells/μL in complete growth medium and seeded in 96-well plastic culture plates (Day 0). On Day 1, 100 μl of lenalidomide (Celgene) (10 μM), 5 FU (10 μM), combination of lenalidomide and 5FU or irrelevant vehicle were added to the cells. On Day 3, cell viability was assessed by quantifying the ATP content by means of a luminescence assay (CellTiter-Glo, Promega). All measurements were recorded with a GloMax 96 Microplate Luminometer (Promega). Growth inhibition at each drug concentration was normalized to vehicle-treated cells.

### Statistical analysis

The means and standard errors were calculated as appropriate. Three or more groups (tumor samples from mice treated with lenalidomide, 5-FU, Combo and irrelevant vehicle) were compared with one-way ANOVA and post analysis comparisons were carried out among each group with Bonferroni post-test analysis. Curves representing tumor growth in mice treated with lenalidomide, 5-FU, Combo and vehicle-treated controls were compared with two-way ANOVA and post analysis comparisons among each group were carried out with Bonferroni post-test analysis. Combo and vehicle treated controls were compared with unpaired t test. Values < 0.05 were considered statistically significant. The results were analyzed with GraphPad Prism5 software, where the P value has the following meaning: (*) P ≤ 0.05; (**) P ≤ 0.01; (***) P ≤ 0.001; (****) P ≤ 0.0001.

## Results

### Tumor vessel density and pericyte coverage in residual tumor vessels

**Fig. 1:**
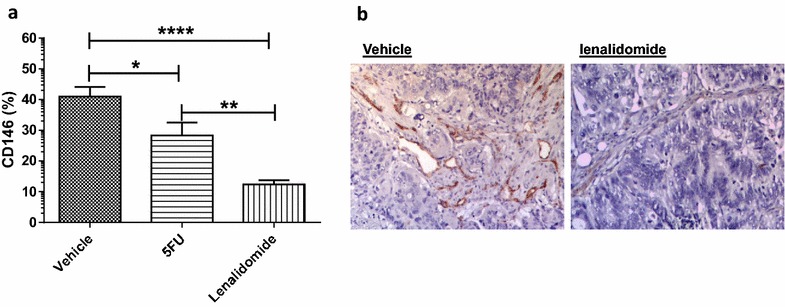
Lenalidomide reduces mCRC vessel density. **a** Lenalidomide significantly reduced tumor vessel density, assessed by CD146 expression, in 5 mice with mCRC tumorgraft compared to the controls treated with irrelevant vehicle or 5FU (p = 0.0001). The cumulative values are presented as mean ± SEM of MOF, the groups were compared by one-way ANOVA. Post analysis comparisons of each group with the control vehicle was performed with Bonferroni post-test analysis (p < 0.05 for 5FU, p < 0.0001 for lenalidomide). **b** The pictures report representative IHC images of CD146 expression in the mice treated with lenalidomide and control vehicle. MOF, microscopic observation fields; (*), P ≤ 0.05; (**), P ≤ 0.01; (***), P ≤ 0.001; (****), P ≤ 0.0001

We explored the activity of lenalidomide on tumor neo-vasculature in a patient-derived tumorgraft model generated from a single KRAS-mutated CRC liver metastasis, propagated in mice. The mice were treated with oral lenalidomide, 5FU or nothing for 28 days after which tumor vessel density in multiple MOF per tumor was analyzed. The mean CD146 expressions were 12 ± 1, 28 ± 4 and 40 ± 3 % in tumors removed from lenalidomide-treated mice (n = 25), 5FU-treated mice and vehicle-treated controls, respectively (p = 0.0001) (Fig. 1a, b).

**Fig. 2:**
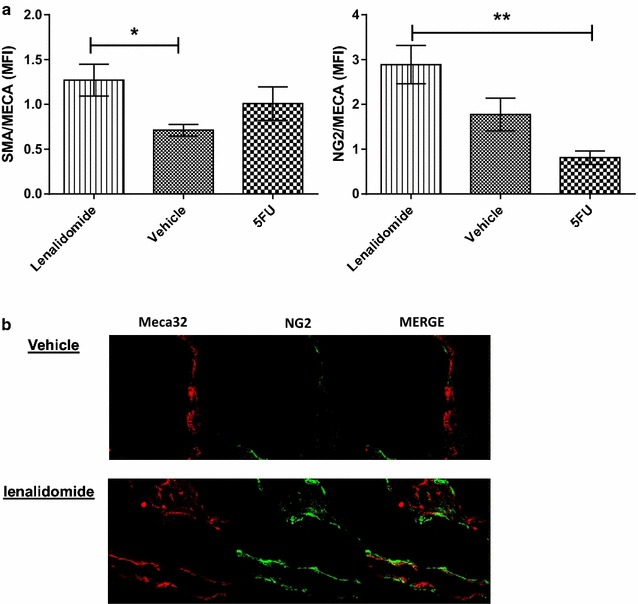
Lenalidomide enhances pericyte coverage of residual mCRC vessels. **a** Lenalidomide significantly enhanced mature pericyte coverage, reported as mean fluorescence intensity (MFI) of α-SMA (p = 0.04) and NG-2 (P = 0.0002) co-localized on vessel areas (MECA32), the results were compared with one-way ANOVA. The values are presented as mean ± SEM of MOF, 3 mice with mCRC tumorgraft per group were included in the experiments. Post analysis comparisons of each group was performed with Bonferroni post-test analysis, resulting in significant p values of lenalidomide against vehicle (*p < 0.05, for α-SMA) and against 5FU (***p < 0.001, for NG2) **b** Representative immunofluorescence picture showing co-localization (*merge*) of NG2 pericyte marker (*green*) and Meca32 endothelial marker (*red*) in tumor samples from mice treated with lenalidomide and control vehicle. MOF, Microscopic observation fields; (*), P ≤ 0.05; (**), P ≤ 0.01; (***), P ≤ 0.001

Pericyte coverage, assessed by expression of pericyte markers NG2 and α-SMA, was significantly enhanced by lenalidomide on residual tumor vasculature compared with untreated controls or those treated with 5FU, p = 0.0002 for NG2 and p = 0.04 for α-SMA respectively (One Way Anova analysis). In fact, the mean expressions of NG2 and α-SMA, each normalized for the vascularized area, were respectively 2.88 ± 0.4 and 1.27 ± 0.2 in tumors explanted for the mice treated with lenalidomide; 1.77 ± 0.4 and 0.7 ± 0.1 for the mice treated with control vehicle, 0.8 ± 0.2 and 1 ± 0.2 for the mice treated with 5FU (Fig. 2a, b).

### Vessel perfusion capability and effect on tumor hypoxia

**Fig. 3:**
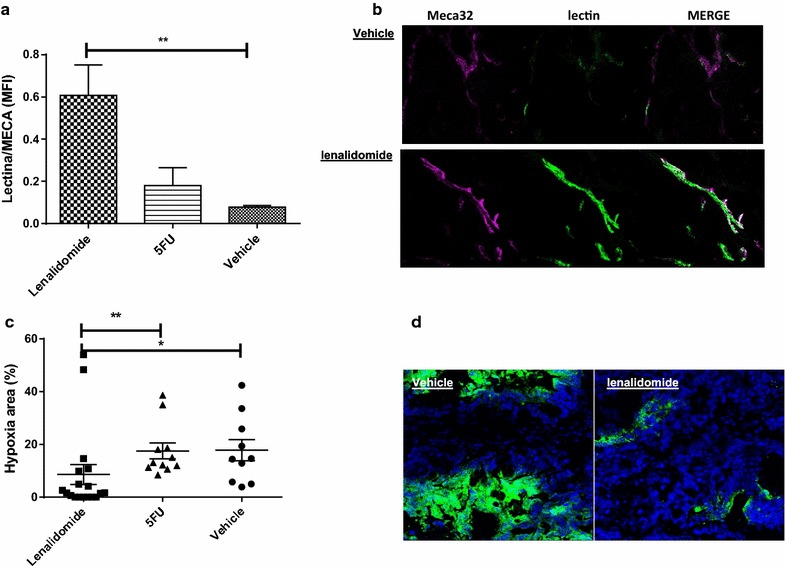
Lenalidomide enhances vessel perfusion capability and decreases tumor hypoxia. **a** Lenalidomide significantly enhanced the perfusion capability of residual tumor vessels reported as MFI of lectin/MECA32 ratio, indicative of vessels with regular perfusion, normalized on total vessel area (p = 0.004 obtained with one-way analysis). The experiments included 3 mice with mCRC tumorgraft per group and the values are presented as mean ± SEM of MOF. Post analysis comparisons (*) of each group to control vehicle was performed with Bonferroni post-test analysis (p < 0.01 for lenalidomide). **b** Representative immunofluorescence picture showing blood perfusion (*lectin in green*) within residual tumor vessels (Meca32 violet as endothelial marker) in mice treated with lenalidomide and vehicle control. **c** Lenalidomide significantly reduced extension of tumor hypoxic areas (p = 0.002) obtained with one-way ANOVA analysis (Kruskal–Wallis). The experiments included 3 mice per group. The* graph* reports values (plus mean ± SEM) of each hypoxic area calculated in vital areas of MOF. Post test analysis comparisons of lenalidomide with control vehicle (p < 0.05 *) and 5FU (p < 0.001**) was performed with Bonferroni post-test analysis. **d** Representative immunofluorescence picture showing hypoxic areas (hypoxyprobe) in sections of tumors from mice treated with lenalidomide and control vehicle. The nuclei are counterstained with DAPI (*blue*). MOF, Microscopic observation fields; (*), P ≤ 0.05; (**), P ≤ 0.01

In order to gain further insight into the normalization effect of lenalidomide, we functionally assessed residual tumor vessels to determine their perfusion capability and consequent effect on tumor hypoxia. Treatment with lenalidomide significantly enhanced the perfusion capability of residual tumor vessels (p = 0.004, One Way Anova,); in fact, the MFI mean values of lectin/MECA32 ratio normalized on the total vessel area, indicative of vessels with regular perfusion, were 0.6 ± 0.1 in tumors explanted from the mice treated with lenalidomide and 0.18 ± 0 in the mice treated with 5FU, compared to 0.08 ± 0.0 observed in vehicle-treated controls (p < 0.001) Fig. 3a, b. Improved perfusion by lenalidomide was associated with the reduction of tumor hypoxic areas (p = 0.002, comparing all treatment groups with one way anova). In fact, the mean extension of the hypoxic areas was 8.6 ± 3.8 % in tumors explanted from lenalidomide-treated mice compared to 17.8 ± 4 % in vehicle-treated controls (p < 0.05) and 17.5 ± 3 % in mice treated with 5FU (p < 0.001); (Fig. 3c, d).

### Vessel normalization enhances chemotherapy activity

**Fig. 4:**
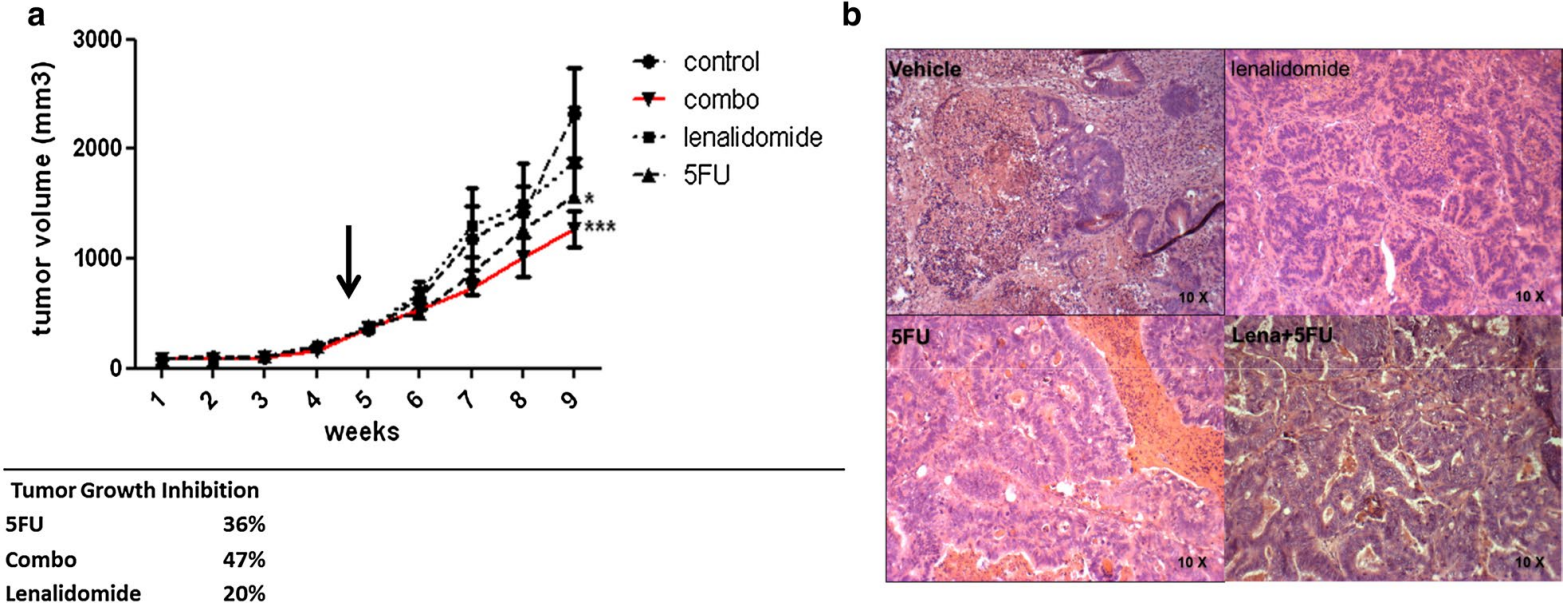
Vessel normalization by lenalidomide enhances chemotherapy activity. **a** The combination of lenalidomide and 5FU significantly delayed tumor growth. The experiments included 8 mice per group. *Curves* with mean tumor volume at each week are reported in the* graph*, the *arrow* indicates the start of treatments. The groups were compared with two-way ANOVA and post analysis comparisons at the end of treatment for each group versus control vehicle with Bonferroni post-test analysis. The percentage of tumor growth inhibition for each group of treatment compared to untreated controls is reported below the curves. **b** Representative histologic images (H&E staining) for each group of treatment. Combination of lenalidomide-5FU was associated with the restoration of tissue organization resembling physiologic colon structures

**Fig. 5:**
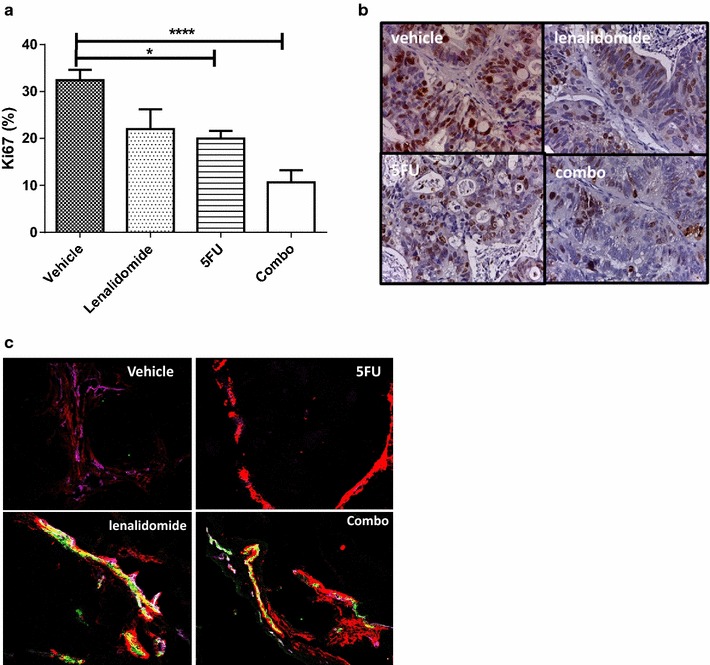
Combination of Lenalidomide-5FU reduced mCRC proliferation index. **a** The combination of lenalidomide and 5FU significantly reduced Ki67 proliferation index in mice with mCRC tumorgraft (N = 6), compared to controls treated with 5FU (N = 5), Lenalidomide (N = 5) or vehicle (N = 6) (p = 0.0002 with one-way ANOVA analysis). The post analysis comparisons of each group to control vehicle was performed with Bonferroni post-test analysis (p < 0.001 for combination therapy). **b** Representative IHC picture of tumor Ki67 expression in each group of treatment. **c** Combination treatment showed effects similar to those observed with lenalidomide for both structural (pericyte coverage) and functional essays (blood perfusion). In the figure is reported a representative immunofluorescence picture showing blood perfusion (*lectin in green*) within residual tumor vessels (MECA 32 violet as endothelial marker, α-SMA in red for pericyte coverage) in mice treated with lenalidomide, vehicle control, 5-FU and combination treatment (*Yellow*/*white* color indicates merging of lectin/MECA32/α-SMA)

We determined whether the vessel normalization induced by lenalidomide could increase the therapeutic index of conventional chemotherapy in vivo. The simultaneous administration of lenalidomide (50 mg/Kg) with 5-FU (20 mg/Kg) for 4 weeks induced a significant delay in tumor growth in CRC xenograft recipient mice compared to vehicle-treated controls or mice treated with single drug lenalidomide or 5FU (P = 0.01, Fig. 4a). Compared to the vehicle-treated controls, the tumor growth inhibition index was 36 % in mice treated with 5FU, 20 % in mice treated with lenalidomide and 47 % in mice treated with the combination of lenalidomide-5FU. A pathological review carried out on tumor samples removed at the end of the experiment revealed that the lenalidomide-5FU combination restored the histological features of a normal colon. The tissue architecture showed physiologic colon crypts, with cells organized as a monostratified epithelium exhibiting reduced polymorphism and polarized nuclei towards the basal membrane. An example of tumor histology following combined lenalidomide-5FU treatment is reported in Fig. 4b. The combination of lenalidomide and 5FU significantly reduced the tumor Ki67 proliferative index (p = 0.0002, comparing all treatment groups by One Way Anova); the mean Ki67 expression was 10.6 ± 2.5 % in mice treated with drug combination, 32.4 ± 2.2 % in mice treated with vehicle control, 19.9 ± 1.6 % in mice treated with 5FU alone, 21.9 ± 4.2 % in mice treated with lenalidomide alone (Fig. 5a, b). Such effect was supported by a vessel normalization effect similar to that observed with lenalidomide for both structural (vessel density; pericyte coverage) and functional aspects (blood perfusion; oxygenation) (Fig. 5c).

Statistical significance (t test) of combination treatment versus vehicle was reached relatively to CD146 expression (22 ± 3 %, p < 0.0001), perfusion capability (MFI lectin/MECA32 = 0.4 ± 0.1; p < 0.0001) and hypoxia (hypoxic area 1 ± 0.5 %; p < 0.001).

### Lenalidomide is devoid of direct tumoricidal activity

**Fig. 6:**
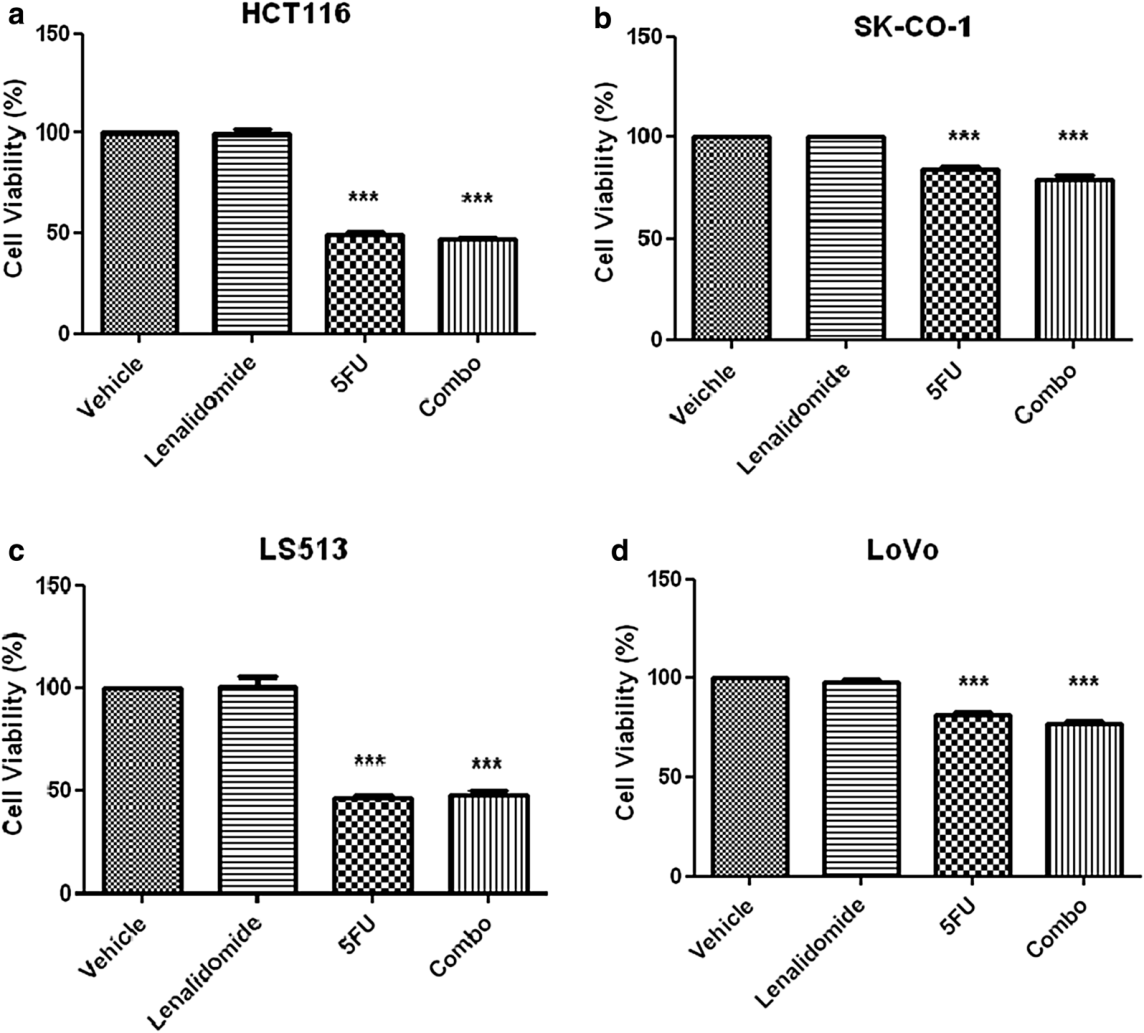
Lenalidomide is devoid of direct tumoricidal activity. Lenalidomide did not have direct cytotoxic activity against 4 different CRC cell lines in vitro (HCT116, LS513, SKCO-1 and LoVo). The* panel* reports data (mean ± SEM) relative to cytotoxic activity against HCT116 (**a**), SK-CO-1 (**b**), LS513 (**c**) and LoVo (**d**) (4 independent experiments, each with 10 replicates). The groups were compared with one-way ANOVA and post analysis comparisons (***) of each group to control vehicle and lenalidomide was carried out with Bonferroni post-test analysis. (***) P ≤ 0.001

The absence of direct antitumor activity observed with lenalidomide in our in vivo CRC tumorgraft model was confirmed in vitro against 4 different CRC cell lines. Treatment with 5FU or combo (lenalidomide-5FU) significantly and similarly killed HCT116, SK-CO-1, LS513 and LOVO CRC cell lines compared to untreated controls or cells treated with lenalidomide only (p < 0.001). A summary of cytotoxicity in vitro against all 4 cell lines is reported in Fig. 6 a-d.

## Discussion

We reported the first evidence that lenalidomide induces normalization of tumor neo-vasculature with improved tissue oxygenation and potential therapeutic benefit in a preclinical in vivo tumorgraft model of mCRC. Lenalidomide is currently used for treating selected hematologic malignancies but as yet little is known regarding its effectiveness for solid tumors. Our findings were obtained in a preclinical tumor xenograft model generated with a liver metastasis from a mCRC patient. Even if potentially limited by its single-patient origin, tumorgrafts may favorable compare with cell line based models for analyzing issues such as maturation and functionality of tumor vessels. Possible strengths are related to the preservation of tumor-architecture, even acknowledging that tumor neo-vessels are mainly murine, and limitation of genetic drift [42].

Lenalidomide has recently been explored in the setting of mCRC with the aim of exploiting its immunomodulation activity for potentiating the ADCC effect induced by cetuximab. Our findings suggest the added value of lenalidomide and broaden its potentialities. In particular our data may further support synergisms with immunotherapy approaches, as the functionality of the vessel network is essential for improving the tumor homing of immune effectors. The conventional angiogenesis inhibition that is expected when treating with lenalidomide was confirmed in our model and the hypothesized “normalization” effect was evident on residual vessels within tumors.

We observed that tumor xenografts treated with lenalidomide in association with 5-FU presented a clear trend towards the (re)differentiation of single cells and global tissue architecture, resembling features of normal colon epithelium, indirectly supporting the beneficial activity of chemotherapy. Several morphologic or molecular parameters may be considered to be indicative of vessel normalization, but ultimately this phenomenon remains a “functional concept” supported by the ability to restore physiologic blood flow and tissue oxygenation. We showed that lenalidomide significantly increased in vivo perfusion capability of residual tumor vessels with regular structures and mature pericyte coverage.

The aim of this study was not to confirm mechanistic molecular mechanisms underlying the activity of lenalidomide as it has already been investigated in previous studies. Based on preclinical evidence, lenalidomide may inhibit VEGF [44, 45], hinder b-FGF-induced endothelial cell cord formation in hypoxic conditions and/or block VEGF-induced endothelial cell Akt phosphorylation, HIF-1α expression and association of the adherent junction proteins in endothelial cells [46]. Instead we focused on the functional implications of this effect. The improvement in tissue oxygenation observed is indicative of normalized blood flow, which may occur to break the vicious circle between the hypoxia-induced factors that promote dysfunctional angiogenesis.

A restored vasculature is important for the efficacy of therapeutic interventions and enhanced activity of chemotherapy with 5-FU was observed. A limitation of our model is its derivation from a single patient’s tumors and may not be representative of all CRCs. Of course, it was not our aim to propose a new therapeutic combination or to demonstrate the known activity of 5-FU against mCRC. The activity of 5-FU was explored only as an experimental tool to confirm the possible functional implications of lenalidomide-induced vessel normalization. Our results provide proof of concept that chemotherapy may indirectly benefit from the activity of lenalidomide on tumor vasculature and the same principle could be applied to other active drugs in mCRC. Moreover, our findings may provide a favorable biological platform for immunotherapy interventions [47]. Functional and restored vessel network is essential for the activity of either approved anti EGFR mAb [48] or other experimental strategies such as novel immune-checkpoint modulators and adoptive cell therapies [26, 49–52]. Ultimately all these strategies rely on the effective trafficking and homing of immune effectors at tumor sites that could consequently benefit from the healthy status of tumor vessels. Furthermore, lenalidomide is endowed with intrinsic immunomodulatory activity, promoting activation of NK and Cytotoxic T cells, potentially fueling a positive biological loop.

## Conclusions

Our findings provide proof of concept that lenalidomide may have indirect, clinically relevant activity in the challenging setting of mCRC. The observed vessel-normalization effect is consistent with the concept of antiangiogenesis as part of a composite therapeutic strategy that benefits from reduced yet functional tumor vasculature. Our data may be useful for carrying out future experimental clinical trials incorporating lenalidomide in order to improve the efficacy of treatments against mCRC. Further preclinical studies are required to in order to determine whether the observed activity of lenalidomide may be extended to other forms of solid tumors and be used in combination with other immunotherapy strategies.

## Abbreviations

mCRC: metastatic colorectal cancer
5FU: 5 fluoruracil
ROI: region of interest
NG2: neural antigen 2
α-SMA: alpha smooth muscle actin
VEGF: vascular endothelial growth factor
b-FGF: basic fibroblast growth factor
HIF-1α: hypoxia-inducible factor 1-alpha
EGFR: epidermal growth factor receptor
NOD/SCID: nonobese diabetic/severe combined immunodeficiency
ADCC: antibody-dependent cell-mediated cytotoxicity
MFI: mean fluorescence intensity
MOF: mycroscopic observational field

## References

[1] Hayes DF (2011). Bevacizumab treatment for solid tumors: boon or bust. JAMA.

[2] Galfrascoli E, Piva S, Cinquini M, Rossi A, La Verde N, Bramati A, Moretti A, Manazza A, Damia G, Torri V (2011). Risk/benefit profile of bevacizumab in metastatic colon cancer: a systematic review and meta-analysis. Dig Liver Dis.

[3] Volz NB, Stintzing S, Zhang W, Yang D, Ning Y, Wakatsuki T, El-Khoueiry RE, Li JE, Kardosh A, Loupakis F (2015). Genes involved in pericyte-driven tumor maturation predict treatment benefit of first-line FOLFIRI plus bevacizumab in patients with metastatic colorectal cancer. Pharmacogenomics J.

[4] Feliu J, Salud A, Safont MJ, García-Girón C, Aparicio J, Vera R, Serra O, Casado E, Jorge M, Escudero P (2014). First-line bevacizumab and capecitabine-oxaliplatin in elderly patients with mCRC: GEMCAD phase II BECOX study. Br J Cancer.

[5] García-Alfonso P, Grande E, Polo E, Afonso R, Reina JJ, Jorge M, Campos JM, Martínez V, Angeles C, Montagut C (2014). The role of antiangiogenic agents in the treatment of patients with advanced colorectal cancer according to K-RAS status. Angiogenesis.

[6] Schwartzberg LS, Rivera F, Karthaus M, Fasola G, Canon JL, Hecht JR, Yu H, Oliner KS, Go WY (2014). PEAK: a randomized, multicenter phase II study of panitumumab plus modified fluorouracil, leucovorin, and oxaliplatin (mFOLFOX6) or bevacizumab plus mFOLFOX6 in patients with previously untreated, unresectable, wild-type KRAS exon 2 metastatic colorectal cancer. J Clin Oncol.

[7] Giampieri R, Scartozzi M, Del Prete M, Fulli A, Faloppi L, Bianconi M, Maccaroni E, Cascinu S (2014). The “angiogenetic ladder”, step-wise angiogenesis inhibition in metastatic colorectal cancer. Cancer Treat Rev.

[8] Hurwitz H, Fehrenbacher L, Novotny W, Cartwright T, Hainsworth J, Heim W, Berlin J, Baron A, Griffing S, Holmgren E (2004). Bevacizumab plus irinotecan, fluorouracil, and leucovorin for metastatic colorectal cancer. NEnglJMed.

[9] Harper J, Moses MA (2006). Molecular regulation of tumor angiogenesis: mechanisms and therapeutic implications. EXS.

[10] Naumov GN, Akslen LA, Folkman J (2006). Role of angiogenesis in human tumor dormancy: animal models of the angiogenic switch. Cell Cycle.

[11] Verheul HM, Voest EE, Schlingemann RO (2004). Are tumours angiogenesis-dependent. J Pathol.

[12] Ellis LM, Haller DG (2008). Bevacizumab beyond progression: does this make sense. J Clin Oncol.

[13] Ellis LM, Hicklin DJ (2008). VEGF-targeted therapy: mechanisms of anti-tumour activity. Nat Rev Cancer.

[14] O’Connor JP, Carano RA, Clamp AR, Ross J, Ho CC, Jackson A, Parker GJ, Rose CJ, Peale FV, Friesenhahn M (2009). Quantifying antivascular effects of monoclonal antibodies to vascular endothelial growth factor: insights from imaging. Clin Cancer Res.

[15] Watanabe M, Boyer JL, Crystal RG (2009). Genetic delivery of bevacizumab to suppress vascular endothelial growth factor-induced high-permeability pulmonary edema. Hum Gene Ther.

[16] Jain RK (2005). Normalization of tumor vasculature: an emerging concept in antiangiogenic therapy. Science.

[17] Klement G, Baruchel S, Rak J, Man S, Clark K, Hicklin DJ, Bohlen P, Kerbel RS (2000). Continuous low-dose therapy with vinblastine and VEGF receptor-2 antibody induces sustained tumor regression without overt toxicity. J Clin Invest.

[18] Huang Y, Goel S, Duda DG, Fukumura D, Jain RK (2013). Vascular normalization as an emerging strategy to enhance cancer immunotherapy. Cancer Res.

[19] Chatterjee S, Wieczorek C, Schöttle J, Siobal M, Hinze Y, Franz T, Florin A, Adamczak J, Heukamp LC, Neumaier B, Ullrich RT (2014). Transient antiangiogenic treatment improves delivery of cytotoxic compounds and therapeutic outcome in lung cancer. Cancer Res.

[20] Fukumura D, Duda DG, Munn LL, Jain RK (2010). Tumor microvasculature and microenvironment: novel insights through intravital imaging in pre-clinical models. Microcirculation.

[21] Dewhirst MW (2009). Relationships between cycling hypoxia, HIF-1, angiogenesis and oxidative stress. Radiat Res.

[22] Rey S, Semenza GL (2010). Hypoxia-inducible factor-1-dependent mechanisms of vascularization and vascular remodelling. Cardiovasc Res.

[23] Krock BL, Skuli N, Simon MC (2011). Hypoxia-induced angiogenesis: good and evil. Genes Cancer.

[24] Yuan F, Chen Y, Dellian M, Safabakhsh N, Ferrara N, Jain RK (1996). Time-dependent vascular regression and permeability changes in established human tumor xenografts induced by an anti-vascular endothelial growth factor/vascular permeability factor antibody. Proc Natl Acad Sci U S A.

[25] Mazzone M, Dettori D, Leite de Oliveira R, Loges S, Schmidt T, Jonckx B, Tian YM, Lanahan AA, Pollard P, Ruiz de Almodovar C (2009). Heterozygous deficiency of PHD2 restores tumor oxygenation and inhibits metastasis via endothelial normalization. Cell.

[26] Hamzah J, Jugold M, Kiessling F, Rigby P, Manzur M, Marti HH, Rabie T, Kaden S, Gröne HJ, Hämmerling GJ (2008). Vascular normalization in Rgs5-deficient tumours promotes immune destruction. Nature.

[27] Stockmann C, Doedens A, Weidemann A, Zhang N, Takeda N, Greenberg JI, Cheresh DA, Johnson RS (2008). Deletion of vascular endothelial growth factor in myeloid cells accelerates tumorigenesis. Nature.

[28] Carmeliet P (2003). Angiogenesis in health and disease. Nat Med.

[29] Jain RK (2003). Molecular regulation of vessel maturation. Nat Med.

[30] Maione F, Giraudo E (2015). Tumor angiogenesis: methods to analyze tumor vasculature and vessel normalization in mouse models of cancer. Methods Mol Biol.

[31] Chanan-Khan AA, Cheson BD (2008). Lenalidomide for the treatment of B-cell malignancies. J Clin Oncol.

[32] Witzig TE, Nowakowski GS, Habermann TM, Goy A, Hernandez-Ilizaliturri FJ, Chiappella A, Vitolo U, Fowler N, Czuczman MS (2015). A comprehensive review of lenalidomide therapy for B-cell non-Hodgkin lymphoma. Ann Oncol.

[33] Gaballa MR, Besa EC (2014). Myelodysplastic syndromes with 5q deletion: pathophysiology and role of lenalidomide. Ann Hematol.

[34] Palumbo A, Hajek R, Delforge M, Kropff M, Petrucci MT, Catalano J, Gisslinger H, Wiktor-Jędrzejczak W, Zodelava M, Weisel K (2012). Continuous lenalidomide treatment for newly diagnosed multiple myeloma. N Engl J Med.

[35] Palumbo A, Freeman J, Weiss L, Fenaux P (2012). The clinical safety of lenalidomide in multiple myeloma and myelodysplastic syndromes. Expert Opin Drug Saf.

[36] Davies F, Baz R (2010). Lenalidomide mode of action: linking bench and clinical findings. Blood Rev.

[37] Motz GT, Coukos G (2011). The parallel lives of angiogenesis and immunosuppression: cancer and other tales. Nat Rev Immunol.

[38] Siena S, Van Cutsem E, Li M, Jungnelius U, Romano A, Beck R, Bencardino K, Elez ME, Prenen H, Sanchis M (2013). Phase II open-label study to assess efficacy and safety of lenalidomide in combination with cetuximab in KRAS-mutant metastatic colorectal cancer. PLoS One.

[39] Gandhi AK, Shi T, Li M, Jungnelius U, Romano A, Tabernero J, Siena S, Schafer PH, Chopra R (2013). Immunomodulatory effects in a phase II study of lenalidomide combined with cetuximab in refractory KRAS-mutant metastatic colorectal cancer patients. PLoS One.

[40] Migliardi G, Sassi F, Torti D, Galimi F, Zanella ER, Buscarino M, Ribero D, Muratore A, Massucco P, Pisacane A (2012). Inhibition of MEK and PI3 K/mTOR suppresses tumor growth but does not cause tumor regression in patient-derived xenografts of RAS-mutant colorectal carcinomas. Clin Cancer Res.

[41] Galimi F, Torti D, Sassi F, Isella C, Corà D, Gastaldi S, Ribero D, Muratore A, Massucco P, Siatis D (2011). Genetic and expression analysis of MET, MACC1, and HGF in metastatic colorectal cancer: response to met inhibition in patient xenografts and pathologic correlations. Clin Cancer Res.

[42] Bertotti A, Migliardi G, Galimi F, Sassi F, Torti D, Isella C, Corà D, Di Nicolantonio F, Buscarino M, Petti C (2011). A molecularly annotated platform of patient-derived xenografts (“xenopatients”) identifies HER2 as an effective therapeutic target in cetuximab-resistant colorectal cancer. Cancer Discov.

[43] Maione F, Molla F, Meda C, Latini R, Zentilin L, Giacca M, Seano G, Serini G, Bussolino F, Giraudo E (2009). Semaphorin 3A is an endogenous angiogenesis inhibitor that blocks tumor growth and normalizes tumor vasculature in transgenic mouse models. J Clin Invest.

[44] Lu L, Payvandi F, Wu L, Zhang LH, Hariri RJ, Man HW, Chen RS, Muller GW, Hughes CC, Stirling DI (2009). The anti-cancer drug lenalidomide inhibits angiogenesis and metastasis via multiple inhibitory effects on endothelial cell function in normoxic and hypoxic conditions. Microvasc Res.

[45] Chauhan D, Singh AV, Ciccarelli B, Richardson PG, Palladino MA, Anderson KC (2010). Combination of novel proteasome inhibitor NPI-0052 and lenalidomide trigger in vitro and in vivo synergistic cytotoxicity in multiple myeloma. Blood.

[46] Calvani M, Rapisarda A, Uranchimeg B, Shoemaker RH, Melillo G (2006). Hypoxic induction of an HIF-1alpha-dependent bFGF autocrine loop drives angiogenesis in human endothelial cells. Blood.

[47] Lanitis E, Irving M, Coukos G (2015). Targeting the tumor vasculature to enhance T cell activity. Curr Opin Immunol.

[48] Ciardiello F, Tortora G (2008). EGFR antagonists in cancer treatment. N Engl J Med.

[49] Naidoo J, Page DB, Wolchok JD (2014). Immune modulation for cancer therapy. Br J Cancer.

[50] Sangiolo D (2011). Cytokine induced killer cells as promising immunotherapy for solid tumors. J Cancer.

[51] Manzur M, Hamzah J, Ganss R (2008). Modulation of the “blood-tumor” barrier improves immunotherapy. Cell Cycle.

[52] Jain RK (2014). Antiangiogenesis strategies revisited: from starving tumors to alleviating hypoxia. Cancer Cell.

